# Medical Papers Presented at the Annual Meeting of the Medical Society of the State of New York

**Published:** 1869-02

**Authors:** 


### Editorial Department.

### Medical Papers presented at the Annual Meeting of the Medical Society of the State of New York.

The Annual Meeting of the Medical Society of the State of New York met according to statute, in Albany, February 2d, 1869, and was called to order by the President, Dr. J. V. P. Quackenbush. Prayer being offered by the Rev. Mr. Larimer, the President proceeded to read his Inaugural Address. He congratulated the Society upon the presence of so many of the permanent members and delegates, and that thus they were able to break away from the routine of professional labor and obtain recreation by mingling in social converse with their associates. He then announced, in most feeling and appropriate manner, the death of Dr. Thomas C. Brinsmade of Troy, and suggested that the Society notice in becoming manner his death.

In representing the condition of the medical profession in the State, he spoke of the medical schools as prosperous, and the system of instruction improved and advancing to greater perfectness, pathology of disease being better *understood* and medicine much better taught than formerly.

He announced the establishment within the last year of two new medical journals: the *Monthly Medical Reprint* and the *American Journal of Obstetrics.* He said: "There are now published in the State, the BUFFALO MEDICAL AND SURGICAL JOURNAL, the *New York Medical Journal*, the *Medical Record*, the *American Journal of Insanity*, the *Quarterly Journal of Psychological Medicine*, the *Medical Gazette*, the *Monthly Medical Reprint*, and the *American Journal of Obstetrics.*"

The various improved instruments and methods of examining disease were enumerated, most of them, however, failing in one respect, the capability of recording their observations and rendering them permanent. The sphygmograph and the dynamograph were exceptions. Another advance was noticed in the present means of representing disease. All former plans were imperfect, till by means of the stereoscope a painting was obtained, which fully equals a view of the specimen itself, which can now be multiplied to any extent, and the instruction derived from it can be enjoyed by him who sees it, almost as well as by him who made the autopsy.

County Societies were urged to hold more frequent meetings, and greater effort to be made to insure the interest of all the members. State Society was deeply interested in this—drew from the County Societies its strength and support.

*Post mortem* examinations were also urged as highly conducive to correct knowledge of disease—said to be much more common in Albany than formerly.

The subjects of revision of the By-Laws and of Renewal of Prescriptions was suggested for consideration, and the address, which was very appropriate and well chosen, was closed by thanks for the honor of being elected presiding officer.

The next paper of importance was a Report by Dr. Hutchison of Brooklyn, upon a case of fracture of femur with great sloughing, where suit for mal-practice had resulted in a verdict for plaintiff for $500. The surgeon was fully sustained by the report, and the sloughing shown to have been in no way connected with tight bandaging, as was claimed by the prosecution.

The Secretary read a communication from Prof. Charles A. Lee, on revision of By-Laws, to the end that the President be hereafter elected by open ballot by members of five years standing.

A very interesting and well prepared eulogium upon the life and character of the late Dr. Thomas C. Brinsmade was read by Dr. George P. Hubbard of Troy.

2D DAY.—Dr. *Robert Newman*, Chairman of Committee upon Results of Consanguineous Marriages, read an elaborate report upon statistics, results, facts and laws. It was a valuable paper and worthy of publication, but not suited to engage the attention of the members during the session of the Society.

Dr. *Henry D. Noyes* of New York, read a paper upon Glaucoma, which attracted the attention of all present. It embodied the symptoms, diagnosis and treatment of the disease, and was illustrated by diagrams and prepared specimens, making the paper both interesting and instructive. It was received by the Society with very manifest satisfaction.

Dr. *Hutchison* of Brooklyn, read his Prize Essay upon Accu-pressure. This is a most complete and elaborate paper and well merits the prize. The modes of making accu-pressure, the effects it produces upon the vessels both of men and animals, the advantages it has over all other modes of arresting hemorrhage, and its safety and reliability, were all shown in a remarkably clear and convincing manner. It is a paper which must be the result of a vast amount of thought and labor, and reflects upon the author the highest credit. We are not prepared to adopt all the conclusions as to the universal applicability, great superiority and perfect safety of accu-pressure, but we are wholly ready to admit the great merit of the Essay, and to commend the careful, continued and well directed observation and experiment of Dr. Hutchison to the universal imitation of the profession.

Dr. *March* of Albany, related a case of spontaneous lithotomy, and showed the stone which had ulcerated its way out of the bladder and presented in the groin. The patient was shown and the sicatrix observed by the members.

Dr. *Wheeler* of Massachusetts, made remarks upon exsection of the head of the femur for caries, as found in hip-joint disease. There was nothing especially new presented in the report, and the specimen shown was such as most surgeons can show in considerable numbers.

Dr. *Corliss* related a case where he had operated for "Ovarian Dropsy" with success, by tapping through the vagina. Patient was nearly well; thought many cases now operated upon by ovariotomy might be cured in the same way; that it might supercede the more capital operation of ovariotomy.

Dr. *James P. White* of Buffalo, did not presume that tapping through the vagina could take the place of, or be substituted for, the removal of ovarian tumor by abdominal section. He related a case where ovariotomy was not regarded as advisable, and had been declined by Dr. Miner, who had previously advised in the case. When presented to him the symptoms were severe and very urgent, and agreeing in the opinion that it could not be successfully removed, he had tapped the tumor through the vagina, with at least great temporary relief. Dr. W. defended the removal of ovarian tumor by operation in all suitable cases, and claimed for it a success as great or even greater than amputation of the thigh after injury, giving as the results of his own experience the past year seven recoveries out of eleven operations.

Dr. *Nathan Bozeman* read a paper upon Vesico-Vaginal Fistula, recommending and explaining the button-hole suture. He seemed to believe that with it success was certain, and without it failure almost equally certain. His paper was well prepared and sustained by fact and argument, but after all, we mistrust no one else was convinced that the button-hole suture possessed any advantages over silver wire or other metal suture secured in the usual way. He presented statistics of the results of his operations, making rather personal comparisons.

Dr. *Bibbins* reported a case of spontaneous recovery from ovarian disease. The report was instructive as showing how nature often institutes modes of recovery which it would appear well for surgeons to attempt to imitate.

Dr. *J. F. Miner* of Buffalo, related a recent case of successful ligature of the external iliac artery, for aneurism, involving the upper portion of the femoral and lower portion of iliac. The point suggested as of special interest was the safe and successful closure of the vessel upon the one side, while the condition of the coats of the arteries were such that aneurism was developing upon the opposite side. The pathological changes which precede and allow of aneurismal tumor were referred to, and the opinion expressed, that little is known concerning the nature or causes of this change.

At this period of the proceedings we were obliged to leave, and as we have not yet seen official report of the transactions of the Society we cannot at present complete a notice of the medical papers brought to the attention of the profession.

The officers of the Society were efficient in the discharge of duty, the President and Secretary uniting in effort to promote the harmony and usefulness of the meeting. The regulation of the exercises and selection of papers to be read before the Society, which is entrusted to the Business Committee, greatly contributes to the interest of the meeting as well as the value of the transactions. This committee, Dr. Sandford Eastman of Buffalo, Chairman, rendered most signal service, and contributed in great degree to the pleasure and profit of the occasion.

Prof. James P. White of Buffalo, was unanimously elected President for the ensuing year. This compliment could not have been conferred upon one more worthy, efficient, or capable, and the profession of Western New York will take pleasure in this recognition of the claims of one of their most distinguished members.

In our next number we hope to give official report of the transactions.

P. S.—The following are the officers for the ensuing year:

*For President*—Dr. James P. White of Buffalo.

*For Vice President*—Dr. George Burr of Binghamton.

*For Secretary*—Dr. William H. Bailey of Albany.

*For Treasurer*—Dr. John V. Lansing of Albany.

